# Correction to: Application of plasma circulating KRAS mutations as a predictive biomarker for targeted treatment of pancreatic cancer

**DOI:** 10.1111/cas.16233

**Published:** 2024-05-29

Lee MR , Woo SM , Kim MK , Han SS , Park SJ , Lee WJ , Lee DE , Choi SI , Choi W , Yoon KA , Chun JW , Kim YH , Kong SY Application of plasma circulating KRAS mutations as a predictive biomarker for targeted treatment of pancreatic cancer. Cancer Sci. 2024;115:1283–1295. doi: 10.1111/cas.16104 38348576 PMC11007020

In the initially published version of this article, there was a discrepancy in the representation of the last digit of the grant number. The correction has no impact on the result and conclusion of the manuscript.

Below is the corrected funding information:

This study was supported by grants from the NCC (Grant number: NCC‐1910192, NCC‐2212470 & NCC‐2310632).

We apologize for this error.

